# Analysis of spatial and temporal trend of hydro-climatic parameters in the Kilombero River Catchment, Tanzania

**DOI:** 10.1038/s41598-023-35105-8

**Published:** 2023-05-15

**Authors:** Onesmo Zakaria Sigalla, Patrick Valimba, Juma Rajabu Selemani, Japhet J. Kashaigili, Madaka Tumbo

**Affiliations:** 1 Nelson Mandela – African Institution of Science and Technology, Nelson Mandera Road, P. O. Box 447, Arusha, Tanzania; 2 Rain Drop Initiative, 109 Regent Estate, Mikocheni, Dar es Salaam, Tanzania, P. O. Box 8703, Dar es Salaam, Tanzania; 3 Department of Water Resources Engineering, College of Engineering and Technology, University of Dar es Salaam, P.O. Box 35131, Dar es Salaam, Tanzania; 4 Department of Forest Resource Assessment and Management, Sokoine University of Agriculture, P.O. Box 3000, Morogoro, Tanzania; 5 Ministry of Water, Water Resources Institute, Off – Sam Nujoma Road, University Road, Ubungo, P. O. Box 35059, Dar es Salaam, Tanzania

**Keywords:** Climate sciences, Environmental sciences, Hydrology

## Abstract

Inadequate knowledge on actual water availability, have raised social-economic conflicts that necessitate proper water management. This requires a better understanding of spatial–temporal trends of hydro-climatic variables as the main contributor to available water for use by sectors of economy. The study has analysed the trend of hydro-climatic variables viz. precipitation, temperature, evapotranspiration and river discharge. One downstream river gauge station was used for discharge data whereas a total of 9 daily observed and 29 grided satellite stations were used for climate data. Climate Hazards Group InfraRed Precipitation was used for precipitation data and Observational-Reanalysis Hybrid was used for Temperature data. Mann–Kendall Statistical test, Sen’s slope estimator and ArcMap Inverse Distance Weighted Interpolation functionality were employed for temporal, magnitude and spatial trend analysis respectively. Results confirmed that, spatially, there are three main climatic zones in the study area viz. Udzungwa escarpment, Kilombero valley and Mahenge escarpment. On temporal analysis, with exception of the declining potential evapotranspiration trend, all other variables are on increase. This is with catchment rates of 2.08 mm/year, 0.05 °C/year, 0.02 °C/year, 498.6 m^3^/s/year and − 2.27 mm/year for precipitation, T_*max*_, T_*min*_, river discharge and PET respectively. Furthermore, rainfalls start late by a month (November) while temperatures picks earlier by September and October for T_*max*_ and T_*min*_ respectively. Water availability matches farming season. However, it is recommended to improve water resources management practices to limit flow impairment as expansions in sectors of economy are expected. Furthermore, landuse change analysis is recommended to ascertain actual trend and hence future water uptake.

## Introduction

### Background

Hydrological studies have been determined by many scholars to be heavily dependent on quantitative data, the quality of these data and how the referred variable behaves over a long period of time^1,2^. Some scholars e.g.,^3–5^ pointed out that data issues will always remain to be a core component of the hydrological sciences. Their character and influence on the way the discipline is practiced may vary through time, but their intrinsic role in understanding and managing water resources and hazards, as well as in developing sound water policies dictates their continuing importance^3^. These data issues as captured by^6^ includes how technology informs surface and subsurface data properties at different spatial and temporal scales, the relative value of traditional hydrological observation vs soft data (a qualitative observation from lay persons, data mining etc.) and extracting information from available data on human and water systems in order to inform the building process of socio-hydrological models and conceptualization. The foundation block on ultimate policy decision on Water Resources Management (WRM) lies predominantly in the understanding of the trend of hydrologic variables and how they interact and relate to each other or the opposite of it^7,8^.

Experience in Africa is that monitoring stations are sparsely scattered, time series data have huge gaps and there is occasional accuracy in data collection^9,10^. The aftermath of this is the limited understanding of changes or variability of climate variables and its implication to water availability at basin scale. It is common knowledge that, the general alteration in climatic conditions directly affect the hydrologic cycle which may be observed in the form of variability in intensity, timing, or depth of hydro-climatic variables^11^. Identifying the trend of large-scale climatic circulation, and its manifestation to local/basin-scale, provides insight into understanding the hydro-climatological process chain^12^. Scientists all over the world are trying to understand the behavior of climate and hydrology through the years in view of the increasing anthropogenic and climatic influence^13^. A good example of these studies and their benefits is presented by^14^ who analyzed the seasonal time series of discharge and sediment load in several tributaries of Yangtze River, China and reported a remarkable alteration in the hydrological parameters, thereby projecting reasonable concern about flooding and water scarcity in different regions of Yangtze River basin.

In an attempt to establish a better understanding of hydrological regime of the study area, scholars have researched different areas of hydro-climatology. This include but not limited to environmental flows assessment^15^, hydrological modelling^16^, groundwater studies e.g., aquifer transmissivity study^17^, the land-use land-cover analysis^18^, evapotranspiration study^19^ and soil mapping studies e.g.,^20^. As pointed out by^21^ in his recollection of decades of research experience in sub-Saharan Africa, he noted the need for locally generated and shaped hydrological modelling. In a quest to contribute to the body of scientific knowledge, this paper intends to examine the spatial and temporal trend of hydro-climatic variables employing Sen’s slope and Mann–Kendall (MK) non parametric statistical test^22^ and ArcMap Inverse Distance Weighted (IDW) Interpolation functionality^23^. The selection of these non-parametric statistical tests was guided by preference accorded to them by the World Meteorology Organization on its superiority in handling data and performing trend statistical analysis^24^. They also have the following advantages^25^ (a) they are considered to work with datasets with more substantial variances (b) they can be useful for interval, ordinal, nominal, and ratio data; (c) the test is not affected, even if the deviation of the dataset is extreme (d) the test can be successfully applied to the skewed dataset. On the other hand, IDW was selected due to its simplicity and highly acceptable results in performing interpolation of climate spatial data^26,27^. Similar methods have been used in Tanzania and elsewhere and provided good results e.g.,^28,29^.

In the current study, the variables under consideration were: temperature, precipitation, evapotranspiration and river discharge for selected sub climatic zones in the catchments. In this regard, evapotranspiration was estimated using Thornthwaite method^30–34^. This is not only because of its being economical on data needs (temperature only) but also it doesn’t suffer regionalization issues which is the case for methods such as Penman–Monteith whose constants are empirical and vary from place to place^31,35^. In addition, when only air temperature data are available, Thornthwaite, Hargreaves and the Blaney–Criddle are the most recommended approaches for estimating evapotranspiration^36^. This endeavor will aid in putting robust and integrated planning framework for different actors in Kilombero River Catchment (KRC) where the Government of Tanzania (GoT) has already laid down multiple water thirsty development plans^37,38^ that are bound to impact one another if business as usual continues^39,40^. Selection of Kilombero catchment was based on the fact that, it presents a better water-energy-food (WEF) nexus in the context of challenges and priorities of a developing nations such as Tanzania. The catchment is center to expansion of food production through Southern Agricultural Growth Corridor (SAGCOT)^41,42^, Its flow contributes to over 60% of water needed for government’s flagship hydropower project i.e., Nyerere project (formerly Stiegler’s project) that is set to generate about 2000 MW of power^37,43,44^. In addition, the flooding and recession of the river benefits species and downstream communities and sustains the largest mangrove forest ecosystem in east Africa^44,45^.

An understanding of pattern and interrelation of key variables is crucial in the context of climate change in order to identify and evaluate current and future uncertainties and risks^8,46^. KRC for instance is characterized by pronounced wet and dry seasons^16,47^ that shapes agriculture as a major livelihood activity and other national scale projects e.g., hydropower generation. There is a spatial aspect of benefits and consequences that must be considered here as increased water access is likely to have positive local impacts but negative downstream consequences because of decreased flows^44^. This spatial variability triggers competition over water and land use conflicts within the study area, at national and regional scale^48^. As such, conflicts emerge between and within different sectors, individual users, communities and ecosystem. These conflicts includes but not limited to competition over little water^49^; human-wildlife conflict^50^ and general conflicts of the use of land and biodiversity resources^51^. To make matters worse, as^52^ emphasised, one important thing to remember about water is that no alternative resource exists for most of its uses. Hence an understanding of long-term trend of variables comes in handy in strategic planning and pre-emptive responses to the said conflicts.

However, as pointed out, there is a lack of research effort that attempted to directly link climate change and variability with river discharge. In this regard and as introduced above, the objectives of this paper are to (1) Quantify and analyse basin-scale temporal and spatial trends in hydro-climatic parameters and (2) Contribute to understanding the potential and limitations of KRC basing on the historical trend of hydro-climatic variables in question.

### Hydrology of study area

The Kilombero Valley’s River network originates from the Udzungwa Mountains on the western rift and the Mahenge Mountains on the eastern rift. The three main rivers are Ruhudji, Mnyera and Mpanga that converge in the Kilombero River. Furua is the main tributary from the east, while Kihansi, Ruipa, Lumemo and Msolwa are the main tributaries on the western bank. Lake Kibasila is one of the few natural lakes in the valley followed by the Kihansi Reservoir which was made for hydropower after damming the river in 90s^53^. Although the area of KRC represents only about 20% of the entire Rufiji basin, the catchment contributes about 62% of the total annual average water flow, 13.8 Bm^3^/year^54,55^ as summarised in Table 1.

At higher elevations and relative to the floodplain, the aquifers are small, weathered and fractured basement aquifers with low to medium groundwater potential. The higher water yield potentials are found in aquifers within the alluvial sedimentary sequence, mostly in the valley bottom^17,56^. Due to the sediments, the aquifers in the valley bottom are in general shallow^17,56^. The recharge to groundwater comes mostly from rainwater infiltration and to a lesser extent from rivers and lakes. Water table levels and precipitation are highly correlated^57^.

**Table 1:** Attributes of different catchments in the RRB^54,57^.

No.	Catchment	Area (Km^2^)	% of Area	Mean annual rainfall (mm)	Mean annual flow (bcm/year)	% of runoff
1	Great Ruaha	85,554	46.6	400–1200	3.3	14.8
2	Kilombero	40,430	21.9	1000–1800	13.8	62.2
3	Luwegu	26,300	13.8	800–1400	4	18.0
4	Rufiji	27,160	17.7	650–1100	11.1	5.0
5	Total	183,791	100		22.2	100

### Climate

The climate in the Kilombero sub-basin is highly variable between the highlands and the lowlands with mean annual rainfall varying from 1100 to 2100 mm^16,47,58^. The highest rainfall (1500–2100 mm) occurs in eastern Mahenge and Central Udzungwa Mountains (which are drained by the Mpanga and Kihansi Rivers) and the low altitude southwest plains^16,47,58^. The lower lying plains of Kilombero receive about 1200 to 1400 mm of rain annually. The largest part of annual rainfall (80–90%) occurs during the rainy season between December and April, while the period from June through September is relatively dry with typical monthly amounts below 10 mm, except in the Udzungwa Mountains^16,47,58^.

While the lowlands are warmer with an annual mean daily temperature of 24 °C at Ifakara, the highlands are cooler with annual mean daily temperature of 17 °C. December and January are the warmest months with day temperature exceeding 27 °C in the lowlands and 19 °C in the highlands^16,47,58^. July is the coolest month with temperature around 21 °C and 14 °C in the lowlands and highlands, respectively. Other climatic variables experience similar spatial variation. Relative humidity varies from 58 to 85% (with an average of 75%) in the lowlands and from 70 to 87% (with an average of 80%) in the Udzungwa Mountains^16,47,58^. Annual potential evaporation in the Kilombero is estimated at 1800 mm per annum. There are trends indicating increasing temperatures and changing precipitation patterns that are expected to cause increased evapotranspiration, reduced runoff and reduced groundwater recharge^57^.

## Materials and methods

### Study area

**Figure 1:**
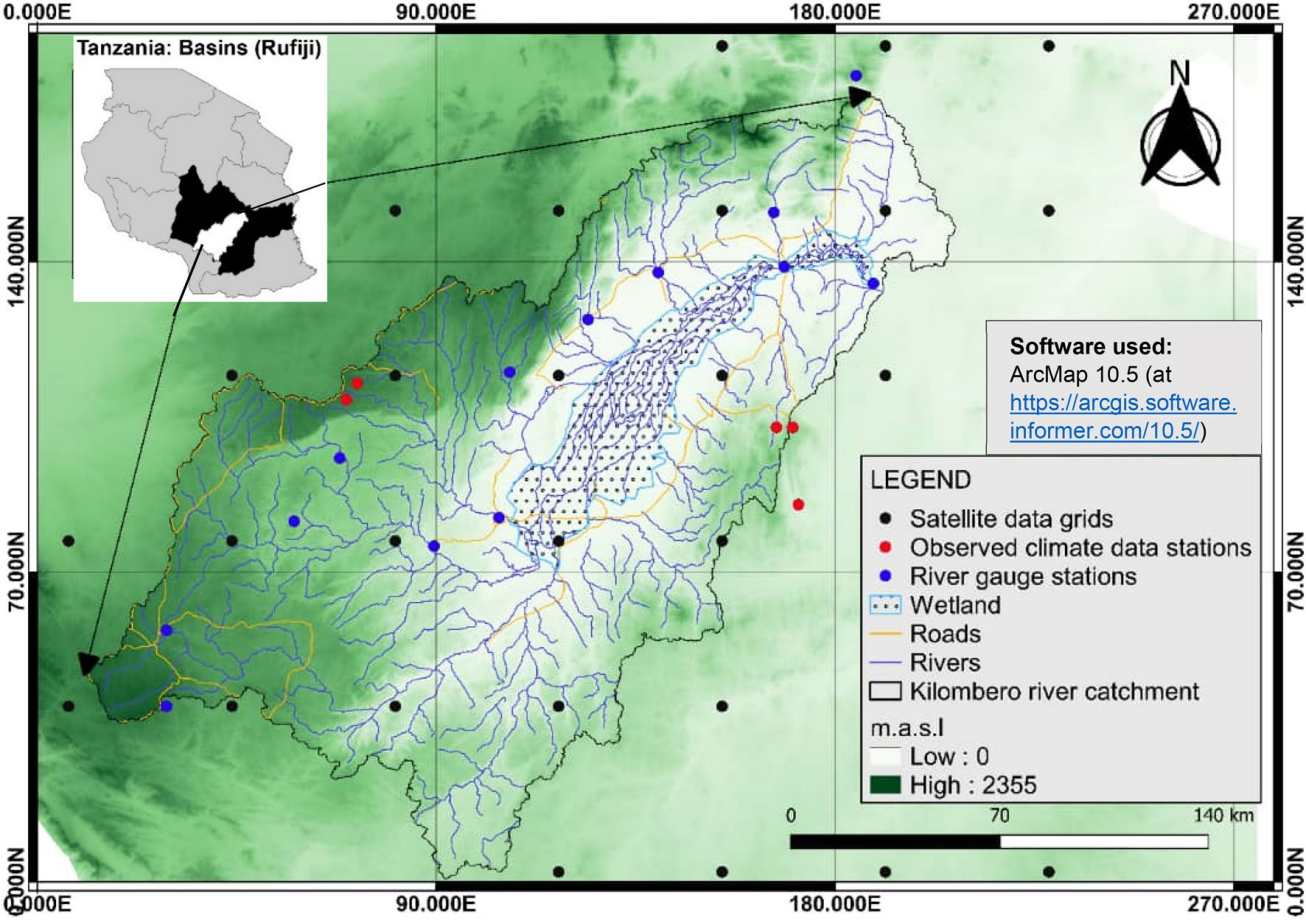
The map of Kilombero River Catchment showing spatial distribution of gauge stations under consideration—modified after^62^.

**Figure 2:**
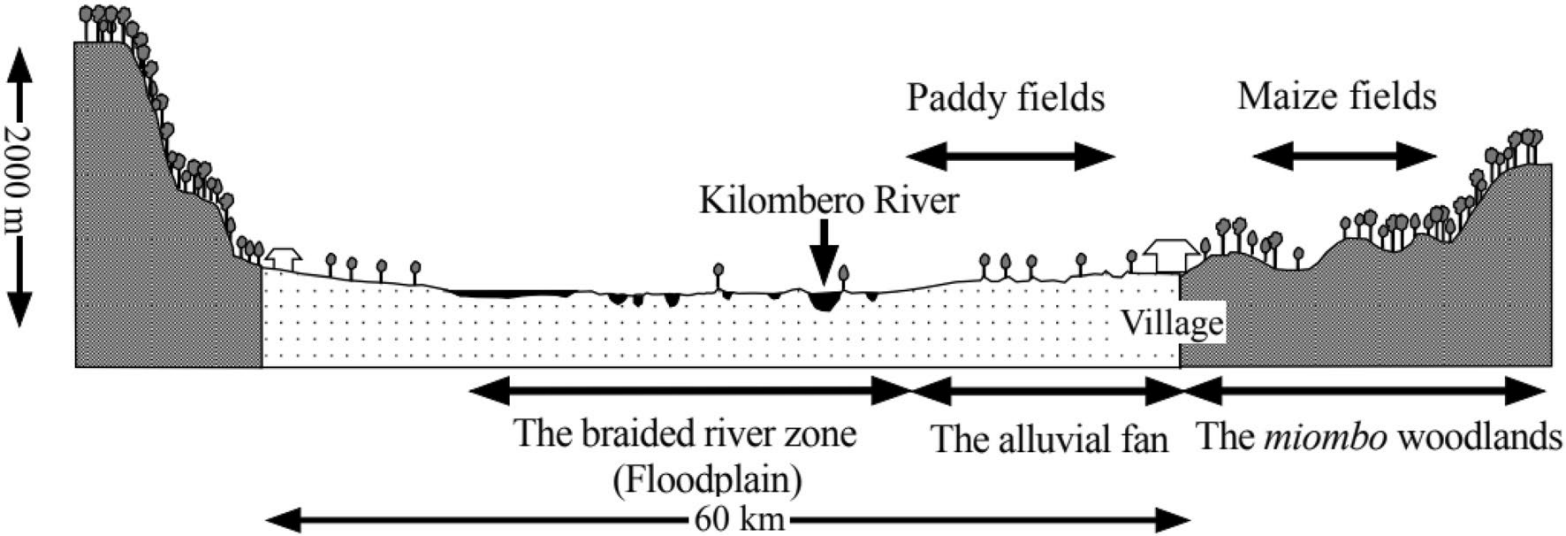
Cross-section of the Kilombero river catchment—adopted from^59^.

This study focuses on Kilombero River Catchment (Fig. 1) that is part of Tanzania’s largest hydrologic basin, the Rufiji River Basin (RRB) spreading across the 177,420 km^2^ (about 20% of Tanzania). The Kilombero River Catchment (KRC) in particular extends between Longitudes 34°00′E–37°20′E and Latitudes 07°40′S–10°00′S and covers an area of approximately 40,000 km^2^^37^. The cross section of the catchment (Fig. 2) is characteristic of a graben structure with Udzungwa mountain ranges and Mbarika escarpments forming the northly and southerly crests respectively while the middle part (the flood plain) forming the trough extending around 1967 km^2^^59,60^. This middle part constitutes one of the largest wetlands in east Africa i.e., Kibasira wetland which is at around 300 m above mean sea level^61^ and most of its area is internationally designated as a Ramsar site for its environmental significance^58^. KRC is the most important catchment in respect of agriculture, energy production, natural resources and flow to RRB^37,58^. Tributaries contributing to KRC are: Lumemo, Luipa, Mngeta, Kihansi, Mpanga, Mnyela, Ruhuji and Furua. Most areas of KRC are situated in the administrative region of Morogoro where its most developed center (Ifakara) is found some 400 km from Dar es Salaam.

### Data and quality control

The study made use of the daily observed data from Tanzania Metrological Authority (TMA) and Rufiji Basin Water Board (RBWB). In addition, to cover missing areas and data, satellite records between 1981 and 2020 were used for climate variables. A total of twenty-nine (29) grided satellite points were used for a better spatial coverage of the area (Fig. 1). The selected satellite data were downloaded from Climate Hazards Group InfraRed Precipitation (CHIRP) for precipitation data whereas Observational-Reanalysis Hybrid (ORH) was used for T_*min*_ and T_*max*_. The selection of these sources were based on validation by previous scholars including^63,64^. In addition, daily river flow discharge data were obtained from an e-flow study by^15^. In addition to other data, a Shuttle Radar Topography Mission (SRTM) digital elevation model (DEM) with a 90 m raster resolution was used to delineate the catchment. Table 2 provides a summary of the data used for this study.

**Table 2:** Data set used in this study including observed and satellite data.

N	Station name	Range	Data type	Resolution	Source
1	Kilombero at Swero	1957–1981	Observed discharge	Daily	RBWB
2	Ifakara MET	2017–2019	Observed precipitation & Temperature	Daily	
3	Mngeta MET			Daily	
4	Echidna	1951–2009	Precipitation	Daily	TMA
5	Kwiro	1933–2009			
6	Mahenge Hosp	1950–2008			
7	Mahenge RF	1993–2019			
8	Matugutu	1961–2015			
9	Ruaha Mission	1950–2002			
10	Mahenge	1993–2019	Temperature	Daily	
11	29 Satellite station grids	1981–2020	Precipitation	Daily	CHIRP https://www.chc.ucsb.edu/data/chirps
			Temperature		ORH https://www7.ncdc.noaa.gov/
			Evaporation		Calculated from Temp
12	DEM	N/A	Elevation	90 m spatial	SRTM https://dwtkns.com/srtm30m/

### Methods

#### Calculation of potential evapotranspiration

Potential evapotranspiration (PET) was calculated by using temperature data based on Thornthwaite formular as discussed in^30–32^. The same is summarised in Eqs. (1)–(4)

1 $$E_{T} = 16L_{a} \left( {\frac{10T}{{I_{t} }}} \right)^{a}$$

2 $$I = \sum\limits_{1}^{12} {i_{n} }$$

3 $$i = \left( {{\raise0.7ex\hbox{${T_{n} }$} \!\mathord{\left/ {\vphantom {{T_{n} } 5}}\right.\kern-0pt} \!\lower0.7ex\hbox{$5$}}} \right)^{1.514}$$

4 $$a = 6.75 \times 10^{ - 7} I^{3} - 7.7 \times 10^{ - 5} I^{2} + 1.792 \times 10^{ - 2} I + 0.49239$$

where *E*_*T*_ is the monthly PET (cm); *T* is the mean monthly air temp (^o^C); *I* is annual heat index in particular year which is taken as summation of monthly heat index values *i*; *L* is average day length (hrs) of the moth being calculated.

#### Statistical trend of time series data

From the dataset explained above, a simple arithmetic mean of variables were calculated to represent higher attitudes in the NE and SW, the lower lying plain in the middle part of the catchment and the mean for entire catchment. This was based on the literature review which indicated that, the study area experienced different climatic conditions in the lower and higher lying terrains.

The non-parametric Mann–Kendall (MK) statistical tests was used with a confidence level of 95%^22,65–67^. Equation (5)–(8) describes the calculations for the selected statistical test involving the key hydroclimatic parameters viz. precipitation, temperature, evaporation and river discharge.

5 $$S=\sum_{k=1}^{n-1}\sum_{j=k+1}^{n}\mathrm{sgn}({x}_{j}-{x}_{k})$$

In this relation, the *sgn* series is defined by Eq. (6) below:

6 $$\mathrm{sgn}\left(x\right)=\left\{\begin{array}{c}+1\dots \dots if\dots \dots \dots ..\left({x}_{j}-{x}_{k}\right)>0\\ 0\dots \dots \dots if\dots \dots \dots ..\left({x}_{j}-{x}_{k}\right)=0\\ -1\dots \dots .if\dots \dots \dots ..\left({x}_{j}-{x}_{k}\right)<0\end{array}\right.$$

where: S is the test statistic, x_*j*_ and x_*k*_ are the sequential variables in a series from *i* =1, 2, … to n − 1 and *j* = *k* + 1, … to n., and n is the length of the sample.

If n is bigger than 8, test statistic S approximates to normal distribution. The mean of S is 0 and the variance of S can be acquired as follows:

7 $$\mathrm{var}(\mathrm{S})=\frac{n\left(n-1\right)(2n+5)}{18}$$

Then the test statistic Z is denoted by Eq. (4)

8 $$Z=\left\{\begin{array}{c}\frac{S+1}{\sqrt{Var(S)}}if\to S>0\\ 0\dots \dots \dots .if\to S=0\\ \frac{S-1}{\sqrt{Var(S)}}if\to S<0\end{array}\right.$$

If Z > 0, it indicates an increasing trend, and vice versa. Given a confidence level α, the sequential data would be supposed to experience statistically significant trend if |Z| > Z1-α/2, where Z1 − α/2 is the corresponding value of P = α/2 following the standard normal distribution.

After determination of increasing, decreasing or no trend by MK test^24,68^, the magnitude of the trend was evaluated by a simple non-parametric slope estimator procedure developed by Sen^69^ as presented in Eqs. (9) and (10) which denotes a liner model that measures change of slope.

9 $${Q}_{i}=\frac{({X}_{j}-{X}_{k})}{(j-k)}for \; all \; k<j \; and \; i=1,..N$$

10 $${Q}_{med}=\left\{\begin{array}{c}Q\left[\frac{\left(n+1\right)}{2}\right], where \; N \; is \; odd\\ Q\left(\frac{N}{2}\right)+Q\left[\frac{N+2}{2}\right], where \; N \; is \; even\end{array}\right.$$

where *Q*_*i*_ is the slope between data points *X*_*j*_ and *X*_*k*_, *Q*_*med*_ is median slope estimator which reflects the direction of the trend in the data.

#### Spatial trend of variables

The spatial analysis was performed under ArcMap 10.5 (at https://arcgis.software.informer.com/10.5/) software spatial analysis function tool. This was performed by Inverse Distance Weighted (IDW) Interpolation as discussed in^70,71^. In this approach the estimation of the value *z* at location *x* is a weighted mean of nearby observations given by Eq. (11).

11 $$\mathrm{z}\left(x\right)=\frac{\sum_{i}^{n}WiZi}{\sum_{i}^{n}Wi}$$

where: Wi = |x – x_*i*_|^−β^ and where *β* ≥ 0 and |.| corresponds to the euclidean distance. The inverse distance power, *β*, determines the degree to which the nearer point(s) are preferred over more distant points. Typically, *β* = 1 or *β* = 2 corresponding to an inverse or inverse squared relationship. The number of surrounding points, *n*, to be included decides whether a global or local weighting is applied. Both parameters *β* and *n* may be fine-tuned by cross-validation. If the point x coincides with an observation location (x = x_*i*_), then the observed value, x, is returned to avoid infinite weights.

## Results

### Spatial trends

**Figure 3:**
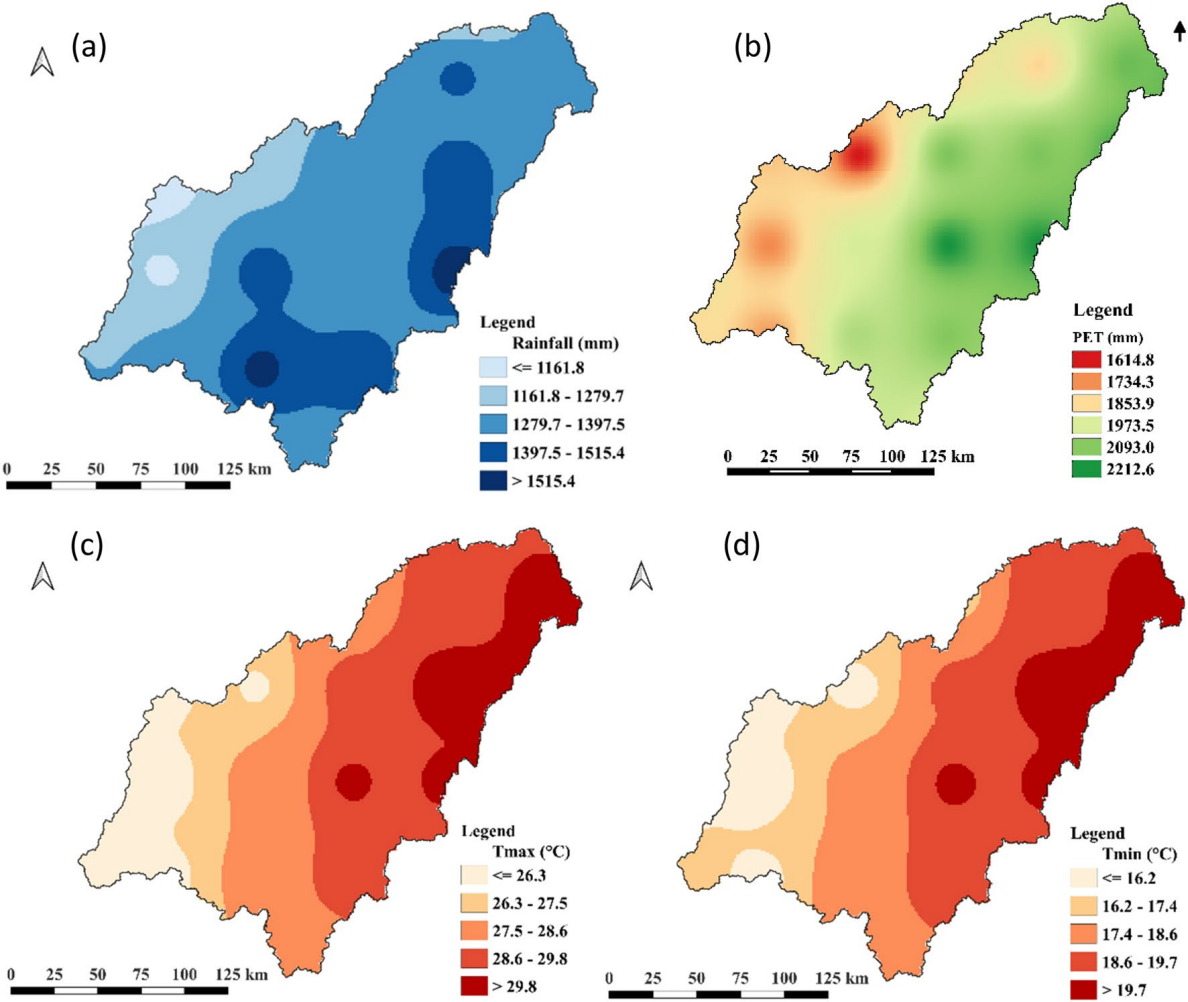
Spatial distribution of climate variables (**a**) Rainfall (**b**) Potential Evapotranspiration (**c**) T_*max*_ and (**c**) T_*min*_.

Due to lack of data, spatial trend was only performed on key climate variable viz. precipitation, temperature and evapotranspiration. This indicates that, the climate of the study area changes significantly as you move from eastern boundary (Mahenge escarpment) through middle part (Kilombero Valley) to western boundary (Udzungwa escarpment) which depicts three main climatic zones. According to this analysis, Mahenge escarpment receive high rainfall and declines in the westerly direction. Similar pattern is indicated by evapotranspiration and temperature variable where values decline with a westerly direction. Most specifically though is the hottest and more evaporation in the NE parts of the valley and coolest and least evapotranspiration at the middle parts of the western boundary of the catchment (the Udzungwa escarpment). Figure 3 gives illustration of these state of climate zones in Kilombero river catchment.

### Temporal trends

With regards to climatic data, the temporal trend analysis was carried out for Mahenge escarpment, Udzungwa escarpment, the Kilombero valley and the average for entire catchment. In addition, hydrology parameter was also assessed using the most downstream station in the catchment i.e., Kilombero at Swero (hydrologic Ref. No.: 1KB17) which is the water volume discharged out of Kilombero catchment after all tributaries and uses upstream.

Furthermore, the long-term observed and satellite data between 1981 and 2019 were used to get descriptive statistics for each individual month to assess seasonality of variables in the catchment, the general long-term statistical trend and the magnitude of increasing or decreasing trend. Results of all variables are presented in subsequent sub sections.

#### Precipitation

##### Seasonality

**Figure 4:**
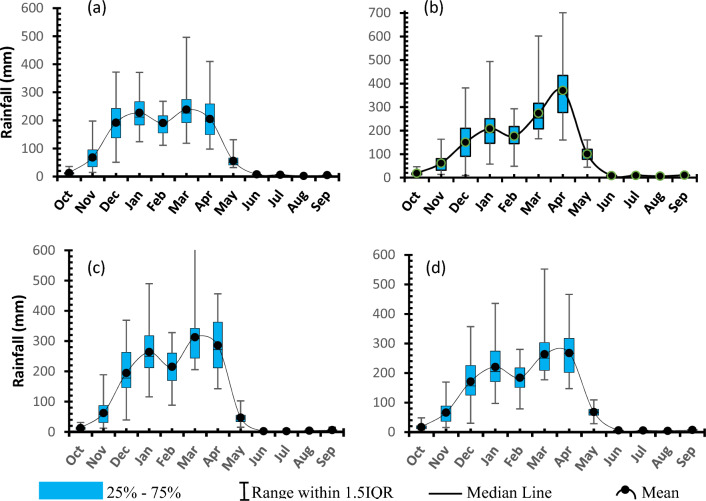
Seasonality of rainfall in (**a**) Udzungwa zone (**b**) Kilombero Valley (**c**) Mahenge zone and (**d**) Average for whole Kilombero Catchment.

Assessment of long-term precipitation data reviled that, in all climate zones and consideration of the entire catchment, a unimodal rainfall pattern is being experienced. The onset is generally around November and it fades away in May. Peak rainfall is experienced in March for the mountainous areas of Udzungwa and Mahenge whereas it is experienced in April in Kilombero valley and under entire catchment consideration (Fig. 4). In addition, all the four considerations show a fairly even distribution of monthly values (median value closer to mean). Furthermore, the data shows that, the interquartile range (IQR) for less rainfall months is small indicating 50% of the data are tightly parked and so the average value is more useful to characterize rainfall of particular month.

##### Long-term statistical trend

**Figure 5:**
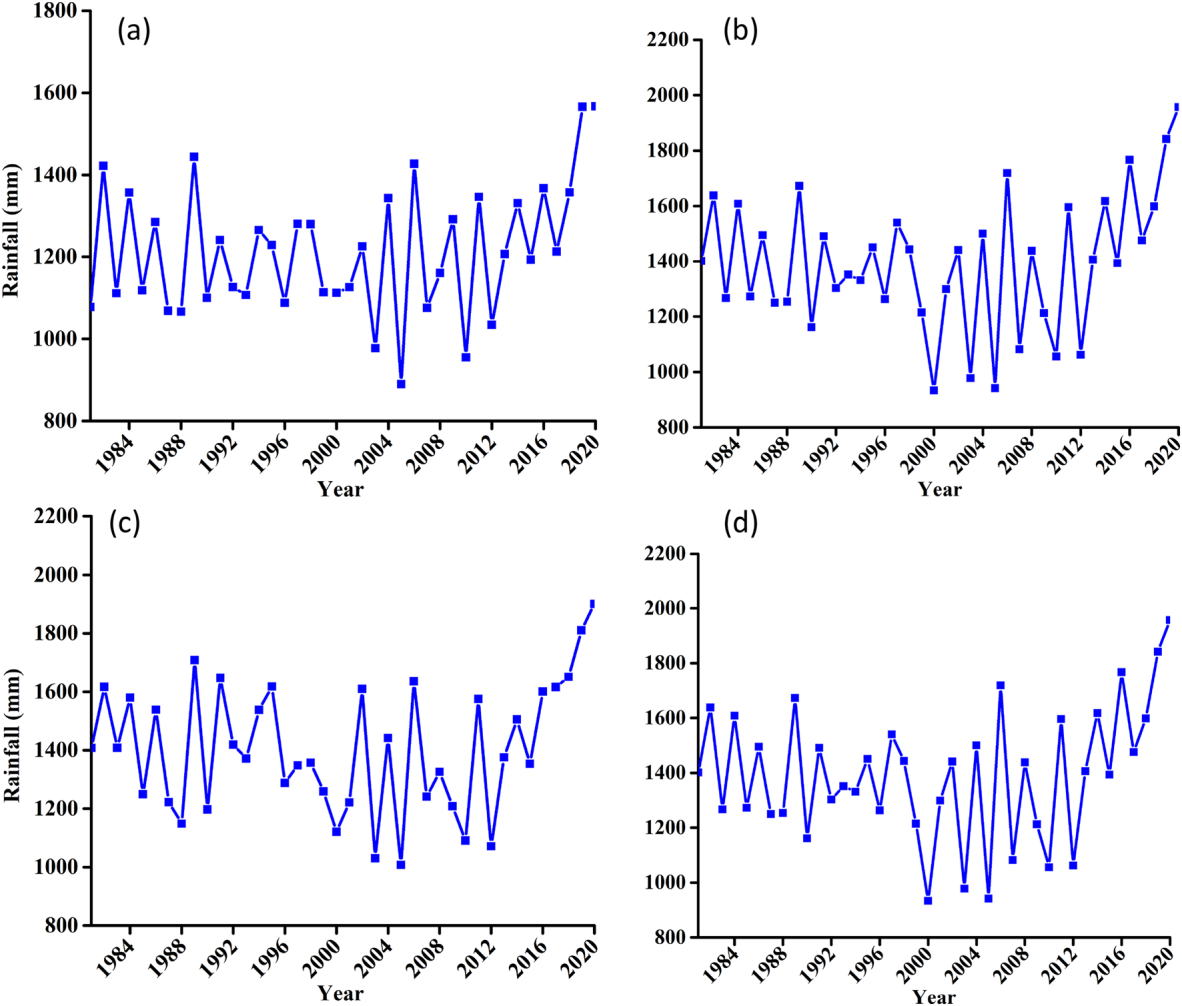
Long-term precipitation timeseries trend for (**a**) Udzungwa zone (**b**) Kilombero Valley (**c**) Mahenge Zone and (**d**) Average for whole Kilombero Catchment.

Results for statistical test of rainfall for the three zones under consideration and the entire catchment are summarized in Table 3. According to the statistical results, there is an increasing trend of rainfall under all four considerations. However, only in Mahenge escarpment the trend is not statistically significant. Observation of graphs as presented in Fig. 5 shows a general increase of precipitation from early 2000s. The magnitude of increasing trend is 2.08 mm/year for the whole catchment average and as presented in the Sen’s slope value for other considered zones.

**Table 3:** Summary of statistical test results for rainfall variable at confidence level of 95% (α = 0.05).

N	Climate zone	Z	Sen's slope	S	Var (s)	Kendal tau	P-value
1	Udzungwa escarpment	1.55	3.73	134.00	7366.67	0.17	0.12
2	Kilombero valley	1.01	4.34	88.00	7366.67	0.11	0.31
3	Mahenge escarpment	0.50	1.65	44.00	7366.67	0.06	0.62
4	Kilombero catchment	0.92	2.08	80.00	7366.67	0.10	0.36

#### Temperature

##### Seasonality

**Figure 6:**
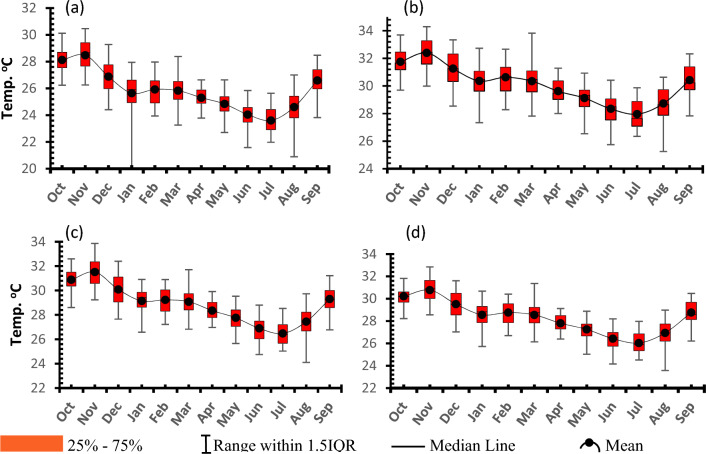
Seasonality of T_*max*_ for (**a**) Udzungwa zone (**b**) Kilombero Valley (**c**) Mahenge Zone and (**d**) Average for whole Kilombero Catchment.

**Figure 7:**
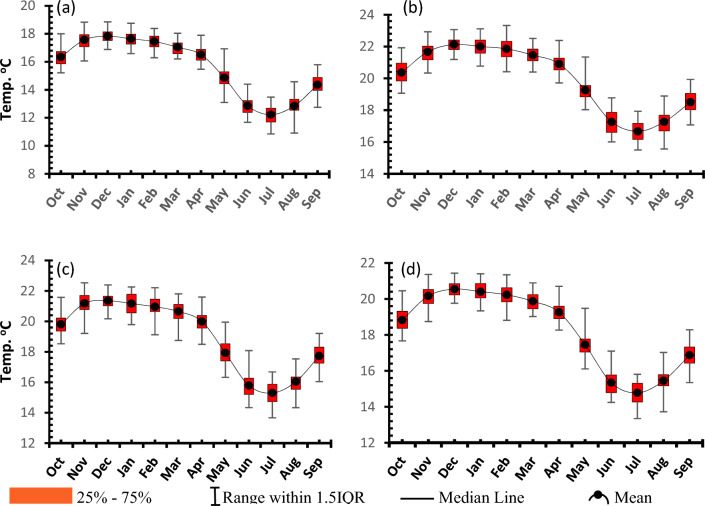
Seasonality of Tmin for (**a**) Udzungwa zone (**b**) Kilombero Valley (**c**) Mahenge Zone and (**d**) Average for whole Kilombero Catchment.

The seasonality of T_*max*_ and T_*min*_ for all the four considerations were assessed using the maximum, mean, median, the lowest and the highest monthly value ever experienced. This shows that, on both cases months of June to August (JJA) are the coolest (Figs. 6 and 7). Whereas for T_*max*_, the months of October to December (OND) recorded the highest values or the warmest (Fig. 6) in the case of the Tmin the high values continued to around March (Fig. 7). As is the case in spatial analysis, Idzungwa experiences the coldest weather, followed by Mahenge and then the middle valley parts especially the NE parts which are the warmest in the study area. In addition, the display of results (Figs. 6, 7 and all sub subsequent seasonality data) helps to not only consider the average value to characterize a particular month and hence season but also actually helps to understand the range especially that of the 50% of data i.e., between 25 and 75% (i.e., Q1 and Q3) hence giving the IQR as a measure of spread of these 50% of data.

##### Long-term statistical trend

**Figure 8:**
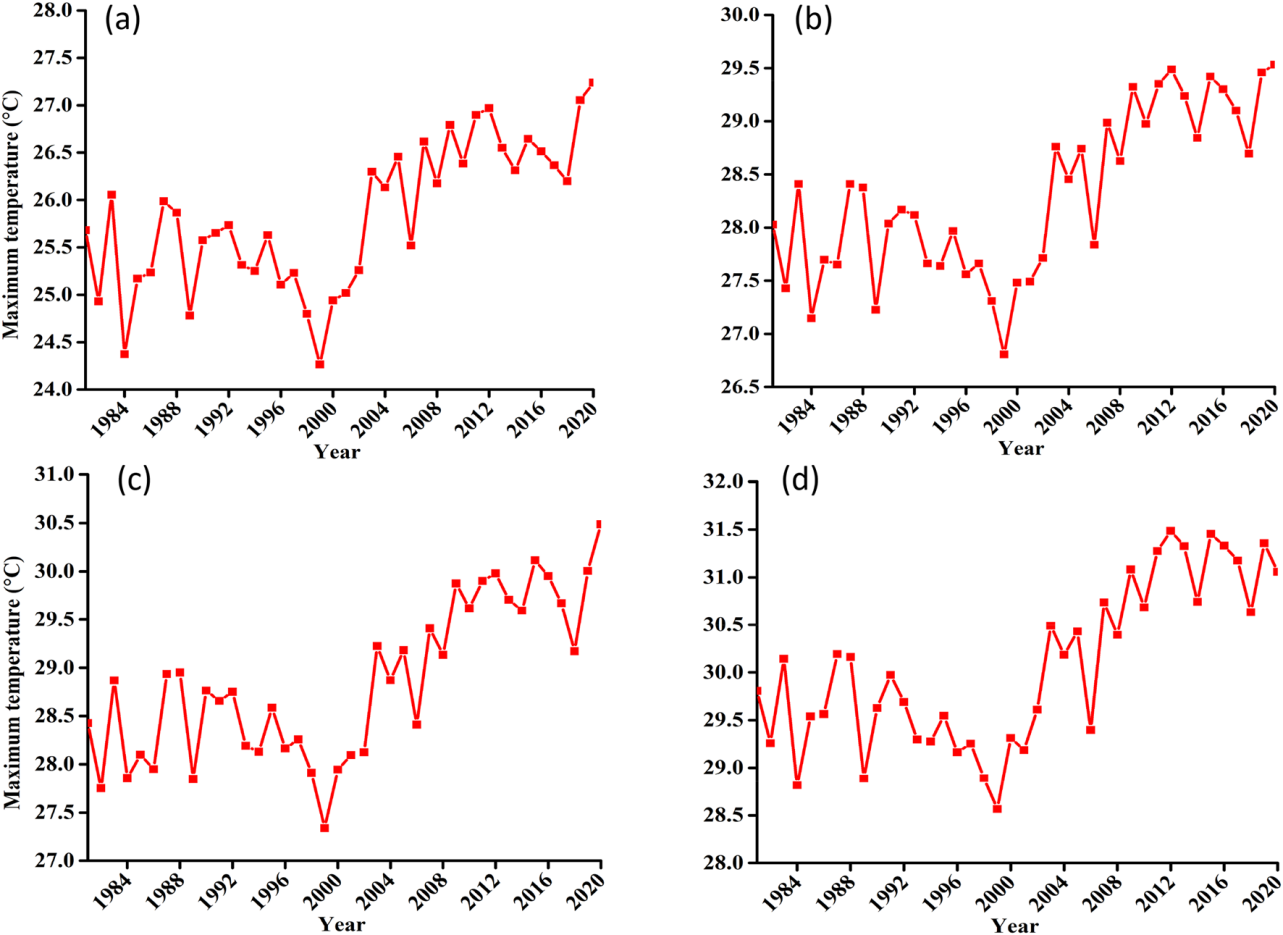
Long-term T_max_ timeseries trend for (**a**) Udzungwa zone (**b**) Kilombero Valley (**c**) Mahenge Zone and (**d**) Average for whole Kilombero Catchment.

**Figure 9:**
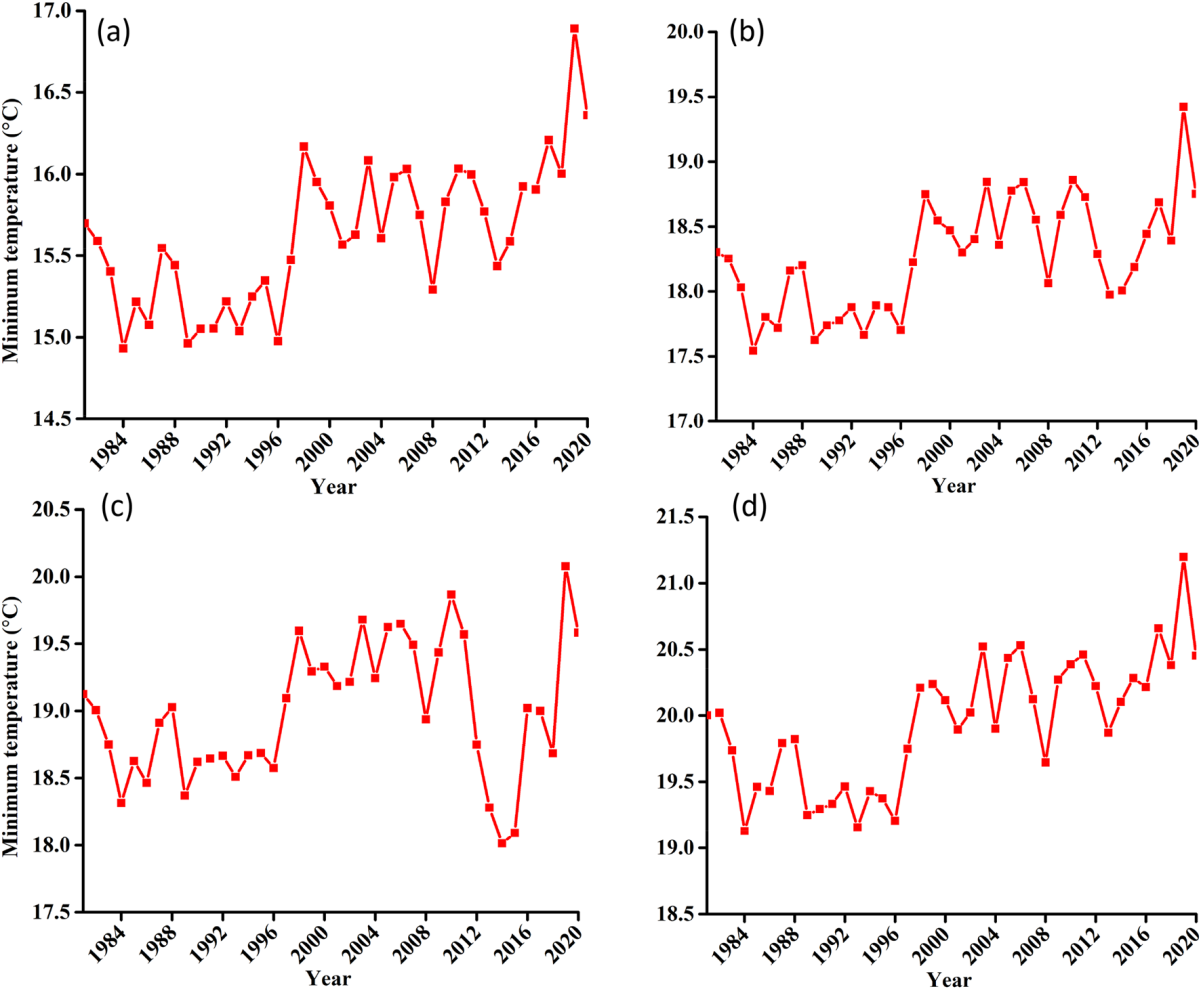
Long-term T_*min*_ timeseries trend for (**a**) Udzungwa zone (**b**) Kilombero Valley (**c**) Mahenge Zone and (**d**) Average for whole Kilombero Catchment.

Tables 4, 5, Figs. 8 and 9 respectively gives a summary and illustration for the statistical results of T_*max*_ and T_*min*_ for the four zones considered in this analysis. These shows that, there is a significant increasing trend of T_*max*_ and T_*min*_ for all the four zones. The magnitude of increasing trend is 0.05 °C/year for all considerations in T_*max*_ and is 0.02 °C/year for Mahenge and entire catchment as compared to an increase of 0.03 °C/year for Udzungwa and Kilombero valley. In addition, whereas T_*max*_ sharp increase occurs in early 2000s, that of T_*min*_ starts at min 90 s.

**Table 4:** Summary of statistical test results for T_*max*_ variable at confidence level of 95% (α = 0.05).

N	Climate zone	Z	Sen's slope	s	Var (s)	Kendal tau	P-value
1	Udzungwa escarpment	4.37	0.05	0.00	0.00	0.00	0.00
2	Kilombero valley	4.39	0.05	378.00	7366.67	0.48	0.00
3	Mahenge escarpment	4.81	0.05	414.00	7366.67	0.53	0.00
4	Kilombero catchment	4.53	0.05	390.00	7366.67	0.50	0.00

**Table 5:** Summary of statistical test results for T_*min*_ variable at confidence level of 95% (α = 0.05).

N	Climate zone	Z	Sen's slope	s	Var (s)	Kendal tau	P-value
1	Udzungwa escarpment	4.39	0.03	378.00	7366.67	0.48	0.00001
2	Kilombero valley	4.58	0.03	394.00	7366.67	0.51	0.00001
3	Mahenge escarpment	2.23	0.02	192.00	7366.67	0.25	0.02606
4	Kilombero catchment	3.72	0.02	320.00	7366.67	0.41	0.00020

#### Evaporation

##### Seasonality

**Figure 10:**
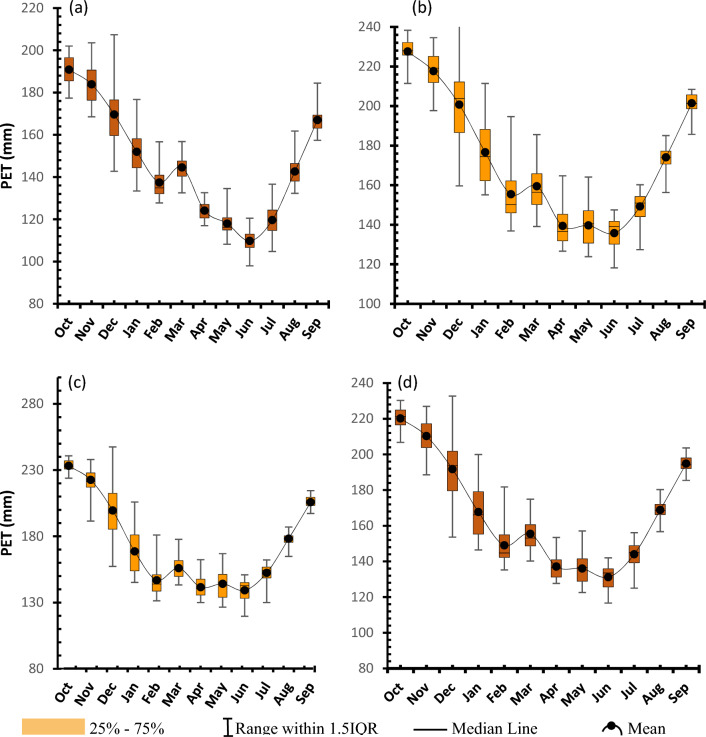
Seasonality of potential evapotranspiration for (**a**) Udzungwa zone (**b**) Kilombero Valley (**c**) Mahenge Zone and (**d**) Average for whole Kilombero Catchment.

As was the case for other variables, descriptive statistics were used to indicate the seasonality pattern of potential evapotranspiration (PET). Results show similar seasonal rise and fall of PET values but in this case the latter comes earlier by a month (i.e., June) in all cases compared to temperature variable (compare Figs. 6 and 7 vs Fig. 10). This might mean that evaporation intensifies when there is enough hotness which happens at latter months.

##### Long-term statistical trend

**Figure 11:**
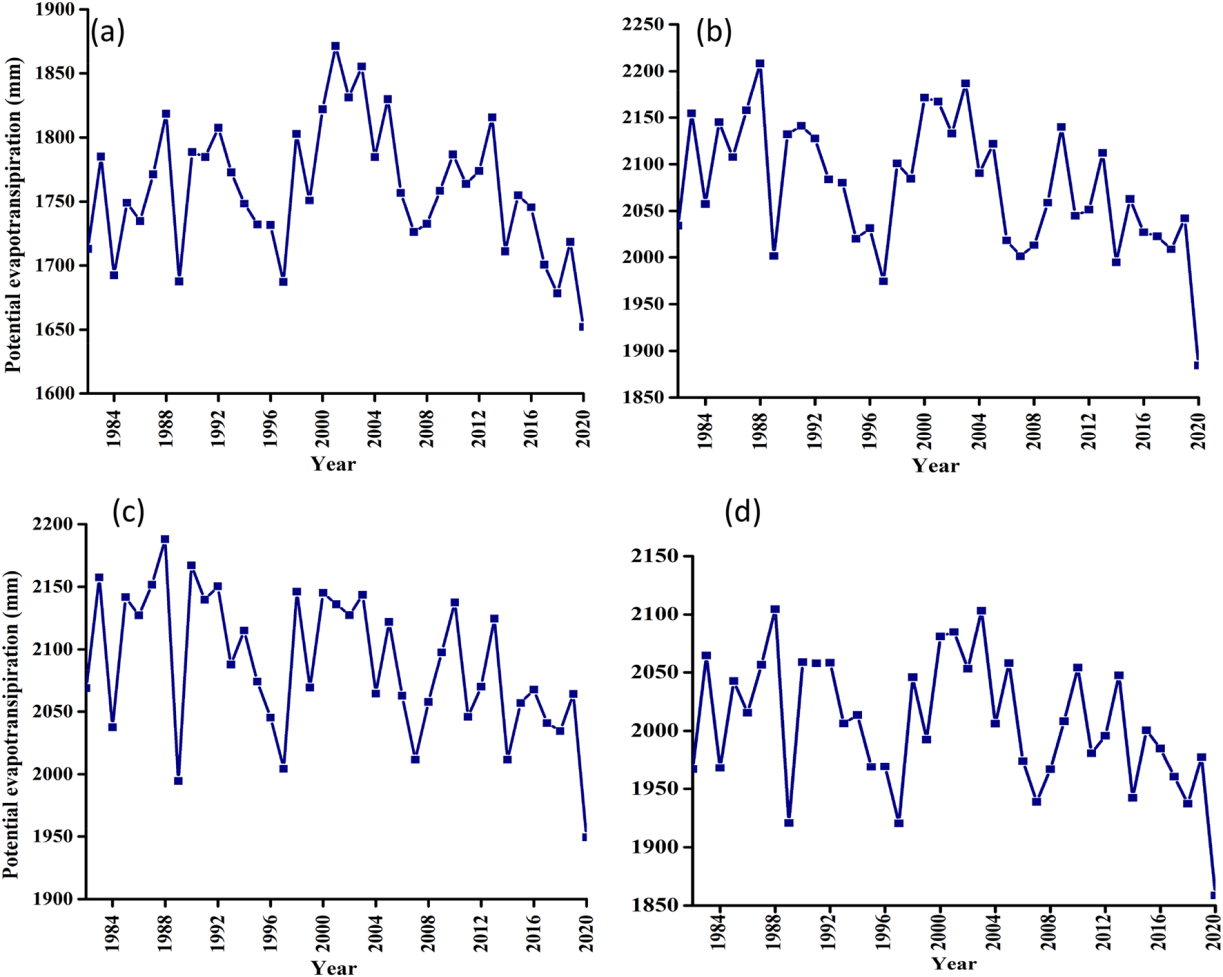
Long-term evaporation timeseries trend for (**a**) Udzungwa zone (**b**) Kilombero Valley (**c**) Mahenge Zone and (**d**) Average for whole Kilombero Catchment.

The test statistical results for evaporation variable are summarised in Table 6 and illustrated in Fig. 11. These results are consistently showing a statistically decreasing trends for all four considerations and an average drop of 2.77 mm/year for the catchment average. Other climatic zones are as shown in Table 6. These results seem to be the exact opposite of all the other variables as a close observation of Fig. 11 indicates the onset of evaporation declination from early 2000s.

**Table 6:** Summary of statistical test results for evaporation variable at confidence level of 95% (α = 0.05).

N	Climate zone	Z	Sen's slope	s	Var (s)	Kendal tau	P-value
1	Udzungwa escarpment	− 0.99	− 0.76	− 83.00	6833.67	− 0.11	0.32
2	Kilombero valley	− 2.73	− 3.03	− 227.00	6833.67	− 0.31	0.01
3	Mahenge escarpment	− 3.24	− 2.33	− 269.00	6833.67	− 0.36	0.00
4	Kilombero catchment	− 2.77	− 1.86	− 181.00	6833.67	− 0.24	0.03

#### River discharge

**Figure 12:**
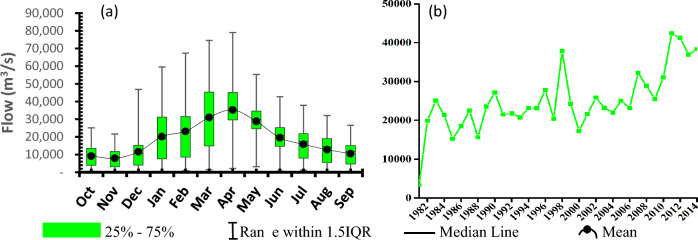
Temporal variability of river discharge for Kilombero at Swero (1KB17) gauge station (**a**) Seasonality and (**b**) Long-term trend.

The long-term trend analysis and seasonality for river discharge were also carried out using the most downstream gauge station i.e., Kilombero at Swero (1KB17). According to this analysis (Fig. 12) the river discharge seems to be increasing with a rate of 498.6 m^3^/s. Other statistical results for this test were as follows: Z-statistics was 4.477 and *p*-value was 0.0000076. In addition to that, seasonality analysis for this station shows a peak flow in April and lowest flow in November. Comparison with rainfall seasonality, shows a quick response between the two variables. The range in seasonal river discharge indicates a highly variable flow pattern.

## Discussion

The unimodal nature of rainfall pattern in the catchment is associated with the position of ITCZ which does not linger over the southern and western to central parts of Tanzania^16,47^. This causes the precipitation centers in the months of SON to be very low to insignificant with substantial rains observed until December whereas the MAM precipitation center being the most dominant^16,72^. Furthermore, the study indicated a general increase in rainfall for all the considered climatic zones. However, perhaps due to finer scale of study, this contradicts with results in other coarser scale studies where a general decrease in precipitation in southern highlands of Tanzania is observed e.g.,^72,73^. Furthermore, in all the four considerations, the results show a consistent delay of rainfall for about a month. Where as hydrologic year for Tanzania starts in October and ends in September, rainfall in the catchment mostly starts in November. This could partly be explained by the distance from Indian ocean (with moist air) travelling to be blocked by Mahenge and Udzungwa escarpments which manifests the orographic rainfall processes that is characteristic of the study area.

On temperature pattern, the study agrees well with other studies e.g.,^72,73^ who indicated a general increasing long term trend for T_*max*_ and T_*min*_ across the country. Furthermore, there is agreement in seasonality of temperature variable which shows months of JJA are the coolest. However, while previously it was indicated that warmer months to be December–January^16,47,58^, our study shows an slight shift with T_max_ being highest between September and December while T_min_ is from October to March. This could be attributed to changing landuse with more bare land being a common phenomenon which may cause temperatures to rise immediately as cold months of JJA which is winter solstice in the southern hemisphere. Furthermore, the assessment of seasonality and long term trend for potential evapotranspiration (PET) indicated that, its highest and lowest in the months of Sept–Nov and May–July respectively. This shows that, temperature and rainfall influences PET in that the seasonality is proportional. This means whenever there is growing temperature and precipitation, environment is more conducive for PET although their peak values are not exactly matching. The long-term trend for PET is however declining which is not consistent with temperature and rainfall trend. This might be explained by other factors such as wind pattern etc. whose evidence is not available and climate stations didn’t measure.

The high precipitation and river discharge in April for the valley part coupled with peak precipitation a month earlier in escarpments means that more water is available for farming activities in the valley part which is most active. At a long-term scale, the comparison of increasing precipitation with declining evaporation means the catchment has a brighter future as there will be more water available for sectors of economy. The increasing trend in discharge for the gauge station in question (most downstream part of the catchment), agrees well with claim that, currently there is enough water compared to uses in the catchment^37^. The increase in discharge is attributed to increase in precipitation which is the major driver influencing river flow^74^. In addition, the peak flow is observed in April which is because of pronounced base flow which peaks in April^16^. By same argument, base flow is expected to be at its lowest in November around which first rainfalls starts and will not have registered any enough water to emerge as baseflow.

## Conclusion and recommendations

In this study, both spatial and temporal trend have been considered for all variables except stream flow in which spatial analysis was not applicable. Analysis shows that, peak agricultural water requirements in the months of MAM coincides well with peak rainfall and river discharge. Furthermore, the declining evaporation in similar magnitude of increasing temperature and the fact that river discharge is also in a rising state means that, available water for sectors of economy e.g., agriculture is plenty if. However, due to improvements in accessibility especially the new trunk road under construction across the Kilombero river catchment, more expansions of farming activities are expected. Therefore, it is advisable to improve water productivity and enhance enforcement of water permits in all sectors to allow sustainable flows downstream.

Together with that, it is recommended to (a) carryout landuse change analysis and trend of expansion of farm lands to ascertain actual water demand for farming activities vs water availability trend (b) Adopt climate smart agricultural practice to improve productivity (c) build capacity of water resources institutions to finetune climate projections and demystify results to appropriate audiences e.g., farmers whose livelihoods depends entirely on how climate behaves and (d) Empower IWRM institutions to enforce the water resources management act.

## Data Availability

Satellite based Precipitation data can be downloaded from Climate Hazards Group InfraRed Precipitation (CHIRPS) at https://www.chc.ucsb.edu/data/chirps where as that of temperature can be downloaded from Observational-Reanalysis Hybrid (ORH) at https://www.ncei.noaa.gov/products/climate-data-records. Observed data are not published online as it is the operational sold data for the hosting institution. Any data access should contact them directly.
